# How well are we translating biofilm research from bench-side to bedside?

**DOI:** 10.1016/j.bioflm.2020.100028

**Published:** 2020-06-05

**Authors:** Kendra P. Rumbaugh

**Affiliations:** a Department of Surgery, Texas Tech University Health Sciences Center, Lubbock, TX, USA; b Immunology and Molecular Microbiology, Texas Tech University Health Sciences Center, Lubbock, TX, USA; c TTUHSC Surgery Burn Center of Research Excellence, Texas Tech University Health Sciences Center, Lubbock, TX, USA

**Keywords:** Biofilm, Anti-Biofilm, Translational research, Biofilm infection

## Abstract

Biofilms are responsible for more than 80% of all chronic infections and represent an enormous medical challenge. In order to meet this challenge, translation research on anti-biofilm approaches is desperately needed. While biofilm research has grown exponentially over the last three decades and provided important details about the mechanisms involved in initiating, maintaining and disrupting bacterial communities, how much of this basic science knowledge has resulted in new therapeutic approaches? In this perspective article biofilm publications, patents, clinical trials and companies were surveyed to ascertain where we stand in translating biofilm research into new strategies to treat and prevent biofilm-associated infections. Overall, the survey data obtained indicate that anti-biofilm research makes up a very small percentage of the total biofilm literature, and the number of patents and clinical studies for anti-biofilm agents is relatively small. However, the forecast for the future of anti-biofilm therapeutics looks promising. Publications on translational studies are trending up and there are a large number of companies selling products marketed to fight biofilm, indicating that there is a significant commercial interest. Researchers can aid in the translational effort by collaborating with clinicians and industry to design and execute clinically relevant pre-clinical studies, which will result in more agents successfully completing clinical studies and entering the market.

## Introduction

Over the last two decades the biofilm field has grown considerably and we have learned a lot about the mechanisms involved in initiating, maintaining and disrupting bacterial communities. Much of this research has extended in to the area of infectious disease as the National Institute of Health has estimated that biofilms are responsible for more than 80% of chronic infections (program announcements PA-03-047, PA-06-537). It is no surprise then that the rationale for many mechanistic biofilm studies is that they will provide important information with which we can combat or prevent biofilm infections, but is this surge of basic science resulting in new therapeutic approaches? The goal of this perspective was to ascertain where we stand in translating biofilm research into new strategies to treat and prevent biofilm-associated infections and to discuss how we can improve these efforts. In order to assess progress in translating biofilm basic science into new products, data from publications, patents, clinical trials and companies was surveyed. This review is based on a presentation I gave at the 2019 Eurobiofilms conference in Glasgow, Scotland.

## Publications

**Fig. 1:**
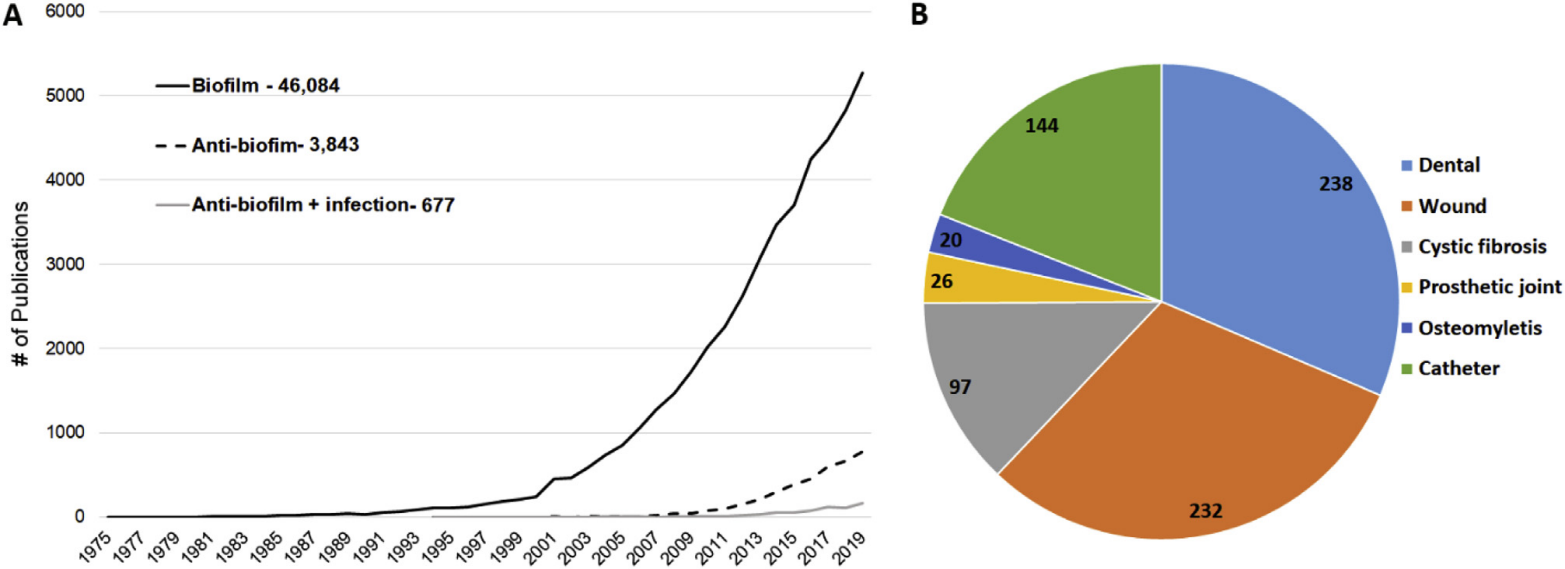
**Overview of the biofilm and anti-biofilm literature.** Search queries were performed in Pubmed using key terms to assess the magnitude of the biofilm and anti-biofilm literature over time (A) and the major areas of disease and infection these studies address (B).

One of the simplest and most straightforward method to gauge the magnitude of research in a specific area is to query the literature. A Pubmed search for papers with the word “biofilm” in the title or abstract illustrates how dramatically publishing on this topic has grown over the last two decades (Fig. 1A). Although the term “biofilm” was found in the abstract of a paper from 1975 [1], the concept didn’t really take hold until the 1990s, and then really gained traction in the 2000s. As of the end of 2019 there have been over 40,000 papers published with a focus on biofilm (Fig. 1A). It should be noted that a query of the word biofilm in all fields yielded 50,513 papers within the same time period; however, by limiting the search to titles and abstracts we are more likely to rule out papers that only tangentially mention biofilm or reference biofilm papers.

In order to determine if this exponential growth in biofilm research has resulted in the development of strategies to combat biofilms, the termed “anti-biofilm” was queried. While several terms have been used in the literature to describe methods of preventing or eliminating biofilms, the term “anti-biofilm” is a term that captures most of the papers on the topic. This is demonstrated in Supplementary Table 1, which shows the numbers of papers resulting from queries of different terms. “Anti-biofilm” or “Antibiofilm” was found in the titles or abstracts of 3843 papers from 1994 (first appearance [2]) to the end of 2019, with 75% in the last 5 years (Fig. 1A). Thus, while anti-biofilm papers represent a small fraction of the total biofilm literature, they appear to be increasing rapidly.

A cursory review of titles from the anti-biofilm literature demonstrates that a large proportion addresses environmental biofilms. In order to specifically identify those studies dealing with medical biofilms, the term “infection” (in the title or abstract) was added to the query and generated 677 papers from 1994 (first appearance [2]) to the end of 2019. If we replace “infection” as a search term with specific medical indications, we can gain an appreciation for the areas where work is being conducted (Fig. 1B). It is not surprising that the majority of these papers refer to dental biofilms, as the dental field was an early adopter of the biofilm concept. Overall though, considering the size of biofilm literature, the fact that only 677 (1.3%) papers primarily address anti-biofilm approaches to medical biofilms, is disappointing. This is especially discouraging considering that it is likely that a majority of the primary biofilm literature discusses the potential importance of their findings to infection.

## Patents

**Fig. 2:**
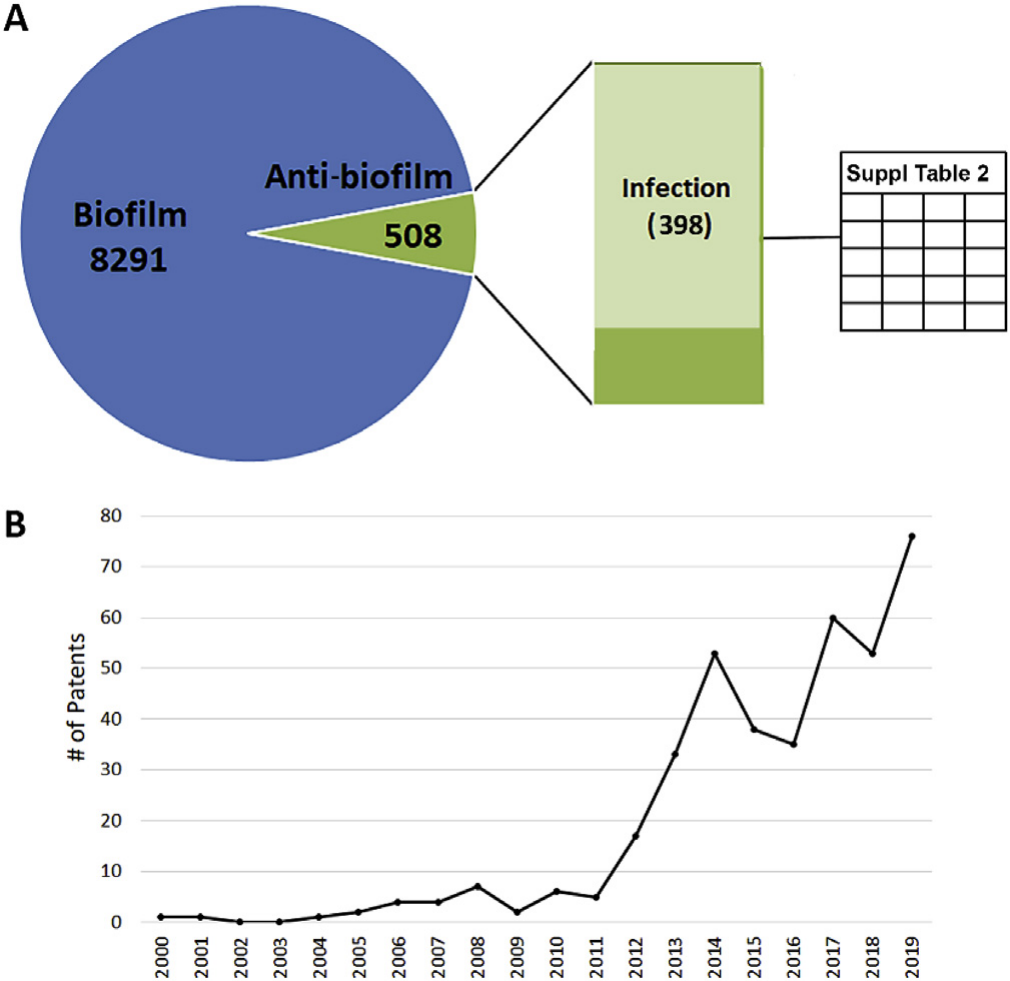
**Overview of biofilm-related patents.** Search queries were performed in United States Patent and Trademark Office (USPTO) patent database using key terms to assess the magnitude of the biofilm and anti-biofilm patents over time.

Another way to assess the level of activity in translating biofilm basic science is to look at the number of patents being issued in the area. For this, I queried the United States Patent and Trademark Office (USPTO) patent database (http://patft.uspto.gov/netahtml/PTO/index.html). From this site full-text patents are searchable from 1976 to the present. Older patents going back to 1790 are also searchable, but only by Issue Date, Patent Number, or Current U.S. Classification, not by keywords. A search for the term ‘biofilm’ in all fields from 1976–Dec. 31, 2019 yielded over 8000 patents, with only 6.1% also referring to ‘anti-biofilm’ (or ‘antibiofilm’). Approximately 78% of these anti-biofilm patents also refer to infection (Fig. 2A). Examination of the titles and abstracts revealed a few patents that clearly focused on agricultural or environmental concerns, so these were not included, leaving a final total of 398 (see Supplemental Table 2 for a complete list). Interestingly, performing the search with Google Patents, which isn’t limited to patents issued in the United States, generated nearly double the number of ‘biofilm’ patents (14,466); however, the number of ‘anti-biofilm’ patents was about nearly identical at 504 and including the term infection reduced this to 298.

The first anti-biofilm patent relating to a medical application was entitled “Metal/thiol biocides” and described chelating bismuth by agents such as a pyrithione or other thiol compounds to form a metal:complexing agent, which has anti-microbial properties [3]. This patent was issued in 2000, and a second patent was issued in 2001 with the same title and inventors, but with a different scope [4]. This is a common occurrence, explaining why several patents have the same title but different issue dates. In this case, the first patent focused on the agents themselves, and the second on methods for administering them. Among the anti-biofilm patents, the largest categories are antimicrobial/anti-biofilm chemicals, including small molecules and natural products. Other common types of patents include anti-biofilm surfaces, devices, and methods for diagnosis and research. Encouragingly, the number of patents has increased dramatically since 2011 (Fig. 2B), which is in line with the increase in anti-biofilm publications (Fig. 1A). Among ‘biofilm or anti-biofilm’ patent holders, companies, universities and individual inventors are included (Supplemental Table 3). The top five are Kane Biotech (13 patents), Harvard University (9 patents), Novabiotics (9 patents), The Katholieke Universiteit Leuven (8 patents) and Microbion (8 patents).

## Clinical trials

Once new products have been patented they must be approved by regulatory bodies such as the U.S. Food and Drug Administration (FDA) before they can be used on humans. For most drugs, devices and procedures, approval will rely on the results of clinical trials that test their efficacy in humans in an unbiased manner. In the U.S., clinical trials for products seeking regulatory approval must be registered on ClinicalTrials.gov, a database maintained by the U.S. National Library of Medicine. Although not necessarily required, many research studies also register their studies on ClinicalTrials.gov if they are using human cells or tissue. Although this site is U.S. based, it is the preeminent site for registering clinical studies and is used worldwide, not just by U.S. investigators. A search of two other large registries, the EU Registry and ISRCTN registry, generated significantly fewer results.

On ClinicalTrials.gov, there are 120 registered trials with “biofilm” listed as a condition or disease and which started on or before Dec. 31, 2019 (first dated 1998, see Supplemental Table 4 for a complete list). To put this in to perspective we can compare the number of biofilm clinical trials to those being conducted on other broad conditions or diseases such as “infection” or “cancer”, which returned 26,970 and 70,442 clinical trials, respectively, within the same timeframe. This is a striking disparity considering that biofilm-associated infections affect more than 17 million people and cause over 550,000 deaths per year, just in the U.S. [5]. In comparison, cancer causes less than 1.7 million new cases and 609,640 deaths per year in the U.S. (2018 estimate from the National Cancer Institute, https://www.cancer.gov/about-cancer/understanding/statistics). Of course, there may be biofilm clinical trials that are not registered, or do not explicitly mention the involvement of biofilm, but it is very unlikely that adding them would make up for the enormity of the shortcomings in this area.

**Fig. 3:**
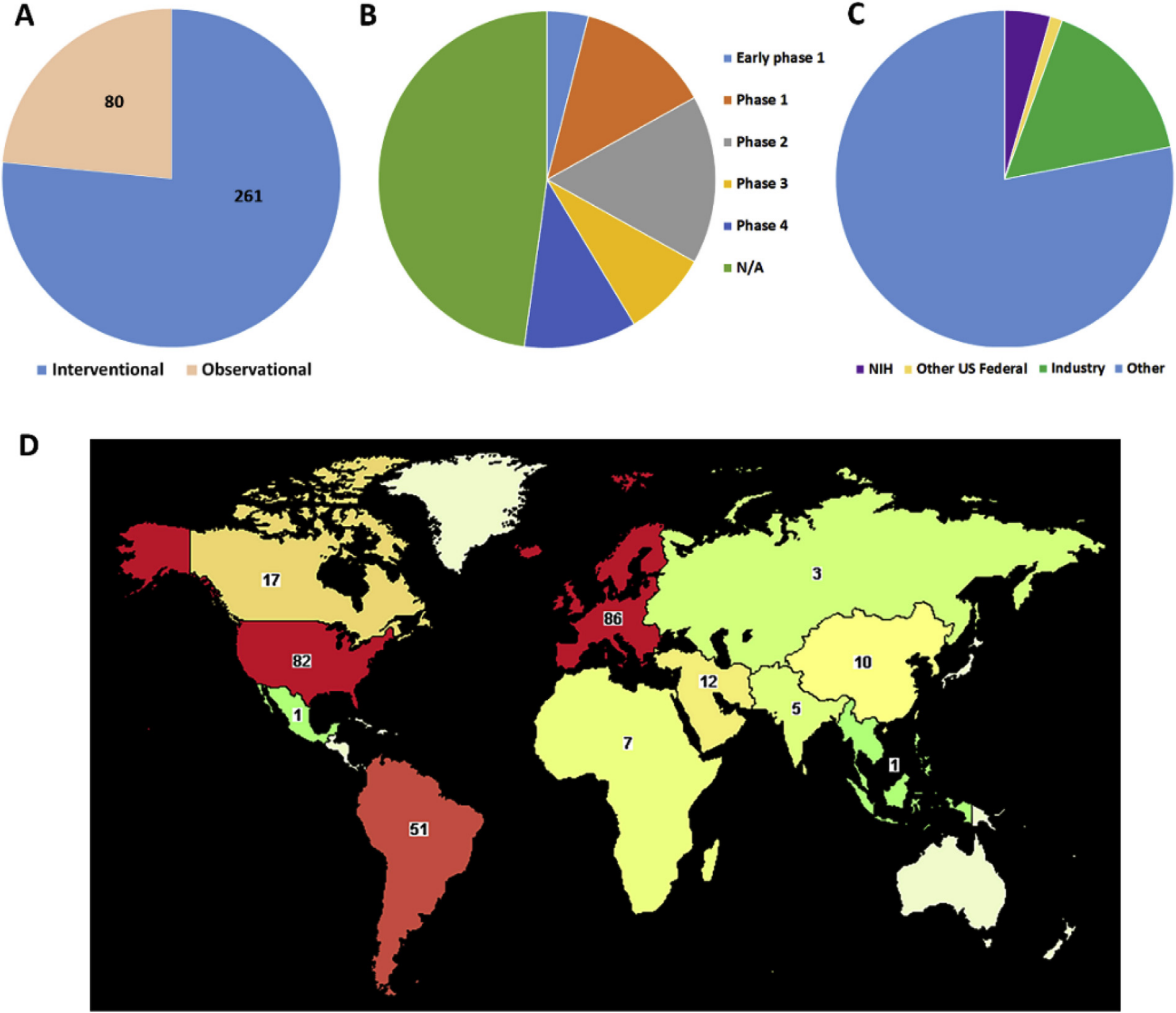
**Biofilm-related clinical studies registered on ClinicalTrials.gov.** Studies are shown by type (A), stage (B), funding type (C), and location (D).

In order to widen the net and include all studies that refer to biofilm, but do not designate it as a specific disease, we can also query all trials that simply list biofilm as a key term. This search results in 341 clinical trials from June 19, 1998 to Dec. 31, 2019. Over 2/3 of these studies were interventional, meaning they are typically prospective and designed to evaluate the direct impact of treatment or preventive measures on disease (Fig. 3A). For example, testing the efficacy of an experimental catheter lock solution to reduce biofilms on catheters and/or reduce the number of catheter-related infections. Of course, interventional studies do not always test new products; they may also compare already approved products or methods to determine which work best to control biofilm infection. Conversely, during an observational study, the investigator is not acting upon study participants. Observational studies are often retrospective and/or epidemiological and are used to assess potential causation in exposure-outcome relationships. For example, a retrospective study to collect patient information in order to determine if obesity is related to development of prosthetic joint infection (PJI) would be observational. While both types of studies can provide important information, observational studies are usually designed to better understand the disease in hopes that new treatments can be developed, while interventional studies are designed to directly determine what treatments work best. Thus, the fact that most biofilm-related clinical studies are interventional is quite promising.

There are different phases of clinical trials that must be accomplished before new products can be approved by the FDA to enter the market. Early phase 1 (formally called phase 0) and phase 1 studies are usually focused on drug safety. Early phase 1 is typically exploratory and involves very limited human exposure, while phase 1 studies usually enroll a small number of healthy volunteers to help determine the drug’s most frequent and serious adverse effects and/or how the drug is processed and excreted by the body. About 1/6 of the biofilm-related clinical studies registered on ClinicalTrials.gov fall into this category (Fig. 3B). Another 1/6 report to be phase 2 studies. These are primarily designed to test the efficacy of a drug/device/procedure on a relatively small group of people who have a certain condition or disease. The participants receiving the experimental agent are typically compared to participants receiving a placebo and/or standard of care agent. During phase 2 adverse events and safety continue to be monitored and studied.

During phase 3, efficacy and safety studies are expanded to include larger numbers of participants, different populations and/or different dosages, or perhaps to evaluate the efficacy of the experimental treatment in combination with other treatments. Lastly, a phase 4 study occurs after the FDA has approved a drug for marketing, so this phase is often referred to as “post-market surveillance”. During this phase, the general public is using the therapeutic and investigators are monitoring long term efficacy and safety. It should be noted that new drugs entering phased clinical trials have a relatively low success rates (14% [6]) and completion of phased trials can be very long (decades) and exorbitantly expensive (5–46 million dollars in the U.S. [7]). Thus, it is not surprising that only about 1/6 of the biofilm-related clinical trials are in these advanced phases (Fig. 3C). Lastly, nearly half of the biofilm-related clinical studies are registered as “not applicable” when it comes to phase. These would include studies without FDA-defined phases, including trials of some devices, behavioral interventions, or very early stage preclinical studies that use human tissue or medical devices extracted from humans.

If we look at the type of research that is being conducted in these biofilm-related clinical trials, we see that the vast majority deal with dental biofilms (Table 1). This is not too surprising considering that dentists were early adopters of the biofilm concept, have access to virtually unlimited human samples, and can conduct human trials relatively easily. On the other hand, it is a bit surprising that other conditions such as cystic fibrosis and peri-implantitis have had so few trials, considering that these involve classic biofilm-associated infections on which so much basic science research has been focused. One unexpected category was ‘digestive’, which mainly focused on mucositis and inflammatory bowel disease. It should be noted that there were many overlapping categories. For example, the list of cystic fibrosis studies completely overlapped with the ‘pancreatic’ list, and all of the ‘endocrine’ studies were also listed under ‘diabetic conditions’.

**Table 1:** Biofilm clinical trial areas^a^.

Stomatognathic diseases • Mouth diseases • Periodontal diseases • Tooth diseases • Dental caries • Tooth demineralization • Dental plaque • Chronic periodontitis • Gingival diseases • Dental deposits Infection • Bacterial infections • Inflammation • Urinary tract infections • Respiratory tract Infections Diabetic conditions	154 105 94 58 35 31 28 24 22 21 99 27 22 14 16 58	Wounds and Injuries • Skin diseases • Foot/leg ulcer Respiratory diseases • Lung diseases o Cystic fibrosis Urologic diseases Digestive system diseases Skin Diseases Vascular diseases Endocrine system disease Fibrosis Peri-implantitis Pancreatic diseases Fibrosis Otorhinolaryngologic diseases	35 16 24 25 17 11 25 22 16 16 13 13 12 11 10 10

a Only disease categories with 10 or more studies were included.

There is a wide spectrum of drugs and devices being studied in these clinical trials for the treatment or prevention of biofilm-associated infections. These include many mouthwash products and solutions to remove biofilm from dentures, different types of toothbrushes, several catheter lock solutions, many different types of catheters, numerous different antimicrobials and wound dressings. While not comprehensive, Table 2, Table 3 provide lists of some of these potential anti-biofilm drugs and devices. Lastly, while the vast majority of interventional studies are designed to test experimental therapeutics, there are some investigations on new ways to detect, diagnose, and test the drug susceptibility of biofilm infections.

**Table 2:** Examples of drugs being tested in biofilm clinical studies.

Dental • Grape Seed Extract ​+ ​nicomethanol fluorhydrate • Inersan (*Lactobacillus brevis* lozenges for gingivitis) • Arestin (minocycline HCl for periodontitis) • Propolis tablet to limit dental biofilm (resinous mixture produced by honey bees) • Xilytol (sugar alcohol) • AMY-101 (complement 3 inhibitor analog) • Nanosilver fluoride • Chlorhexidine • Essential oils • Ferumoxytol (feraheme) • Triclosan • Pomegranate (Punica granatum Linn) • Black and green tea • Rumex acetosa leaf extract (periodontitis prophylaxis) • Methylene blue (methylthioninium chloride, photodynamic therapy) • Colloidal silver nanoparticles • *Lactobacillus reuteri* DSM 17938 and PTA 5289 Catheter, stents • *N*-acetyl cysteine, tigecycline and heparin (catheter lock solution) • Neutrolin (taurolidine, heparin, calcium citrate, catheter lock solution) • Triclosan • Auriclosene irrigation solution (organosulfonic acid)	Lung • Nitric oxide (for cystic fibrosis) • Arikayce™ (amikacin liposome inhalation suspension, for cystic fibrosis) • Cysteamine (for cystic fibrosis) • Inhaled SNSP113 (poly N (acetyl, arginyl) glucosamine for cystic fibrosis) Wounds/Soft tissue infections • VeraFlo with Prontosan benzalkonium gel • Chlorhexidine • Nitric Oxide • Methylene blue (methylthioninium chloride, photodynamic therapy) • BLASTX Gel • serine protease Esp protein to control *S. aureus* wound biofilms • Ceramiseal (topical serum with proprietary blend of naturally occurring lipids, for wounds) UTI/Kidney • Velphoro (sucroferric oxyhydroxide, chewable tablets) • Nitric oxide Eye • NVC-422 (*N, N*-dichloro-2, 2-dimethyltaurine, conjunctivitis) *Helicobacter pylori* Infection • *N*-Acetyl cysteine Vaginosis • *N*-Acetyl cysteine Sinusitis • Colloidal silver nanoparticles • Topical silver colloid • Xilytol

**Table 3:** Examples of devices being tested in biofilm clinical studies.

Catheters, stents, tubes • GamCath Dolphin® Protect central venous catheter (bismuth-containing surface coating) • Ureteral Stent (triclosan releasing) • Insertion of a urinary catheter coated with benign *E. coli* • Radiance™ Clear Sharklet® Silicone Foley Catheter (micro-patterned surface) • M3 MINI CATHETER • Nitric Oxide impregnated catheter • Oxys Catheter (electromagnetic therapy) • CAM (Complete Airway Management) Catheters • Airway medix closed suction system (endotracheal tube) • Vented urinary drainage system • UroShield device (ultrasound acoustic waves) • PE tube with Duckbill Valve (for otitis media) Vaginosis • Vaginal suppository: WO3191 (amphoteric surfactant) • Vaginal suppository: Vagisan® Lactic Acid	Wounds • Procellera (dressing with electrical micro current) • MIST Therapy is a low energy, low intensity ultrasound delivered through a saline mist to the wound bed • Larval Debridement Therapy • Zorflex Activated Carbon Dressing • VAC VeraFlo with Dakins Instillation • VAC Ulta Therapy • Iodosorb (Cadexomer Iodine Gel) • Aquacel® Ag ​+ ​Extra • Epiceram (ceramides, cholesterol and free fatty acids) • PuraPly™ Antimicrobial Wound Matrix (collagen ​+ ​polyhexamethylenebiguanide hydrochloride (PHMB)) • Exsalt SD7, Exsalt T7 Wound Dressings (silver salts) Prosthetic Joint Infection • Ioban (iodine impregnated incisional foil)

As mentioned, clinical trials can be expensive, especially if they enter into late phases. In looking at funders of the 341 biofilm-related studies, it appears that the vast majority of funding is coming from non-federal (U.S.) and non-industry sources. Although it is not clear what the actual funding sources are, they could include private foundations, institutions, and non-U.S. government funders. The latter seems reasonable, given that there are more biofilm studies registered abroad, especially in Europe, than in the U.S. (Fig. 3D). This is also surprising considering that for many other conditions, the majority of research is conducted in the U.S. For example, if we compare to cancer studies again, we see that the majority are registered in the U.S. (41% of all cancer studies worldwide, with Europe second at 23%) and government and industry fund far fewer U.S. biofilm studies than cancer studies (22 and 52% respectively). This suggests that the U.S. has not invested as much attention or money into the biofilm problem as many other countries.

Interestingly, searching clinical trials for the key term anti-biofilm (or antibiofilm) resulted in 6 studies, two of which were not included in the 341 biofilm-related studies discussed above (Supplemental Table 5). These studies focus on a variety of treatments for conditions including dental caries, wound infections, vaginosis, and catheter-associated infections. Encouragingly, two of these are phase 4 studies, including the evaluation of a benzalkonium irrigation solution and gel to treat wounds and *N*-Acetyl cysteine to treat bacterial vaginosis.

## Industry

Lastly, we can assess whether biofilm research is being translated into new treatments by looking to see if products are entering the commercial market. One way to do this is to search for companies in the area of biofilm. There are several search engines for companies, including VentureRadar with which you can search for companies by keyword. Searching by the keyword “biofilm” on this site returns 156 companies, many of which are clearly focused on environmental concerns such as bioremediation, biofouling and wastewater. Examples of the companies in the medical biofilm area are shown in Table 4.

**Table 4:** Examples of companies in the biofilm and anti-biofilm areas.

Company name	Year founded	Location	Website	Products
Biofilm Pharma	2017	France	biofilmpharma.com	Antibiofilm non antibiotics
BioFilm ​Control	2005	France	biofilmcontrol.com	Biofilm testing
BioSurface ​Technologies	1994	USA	biofilms.biz	Biofilm reactor systems
Aequor ​Inc.	2006	USA	aequorinc.com	Anti-biofilm small molecules
Kane Biotech	2001	Canada	kanebiotech.com	DispersinB
Perfectus ​Biomed	2012	UK	perfectusbiomed.com	Biofilm testing
Quorum ​Innovations	2010	USA	quoruminnovations.com	Probiotics
AlgiPharma	2006	Norway	algipharma.com	Alginate technologies
Primal ​Therapies, Inc.	2012	USA	primaltx.com	Selective ​microbial ​metabolism ​regulation ​technology
Agile Sciences, Inc.	2007	USA	agilesci.com	Antimicrobial small molecules
Microphyt	2007	France	microphyt.eu	Microalgae and their products
Bioseka	2011	Lithuania	bioseka.eu	antisense oligonucleotides as antimicrobial agents
PhagoMed ​Biopharma	2017	Austria	phagomed.com	Phage therapy
Lynntech, ​Inc.	1987	USA	lynntech.com	Engineered products
Curza	2013	USA	curza.com	Small-molecules
CamStent	2006	UK	camstent.com	Polymer coatings for medical implants and devices
Gene&GreenTK	2013	France	gene-greentk.com	Enzymatic decontamination and quorum sensing inhibition
Next ​Science	2012	Australia	nextscience.org	Anti-biofilm topical agents
BioVersys GmbH	2008	Switzerland	bioversys.com	Transcriptional regulator inhibitory compounds
Madam Therapeutics	2011	Netherlands	madam-therapeutics.com	Synthetic antimicrobial and anti-biofilm peptides
Selenbio, Inc.	2004	USA	selenbio.com	Selenium coated surfaces
ISurTec	unknown	USA	isurtec.com	Anti-biofilm surfaces
Microbion Corporation	2000	USA	microbioncorp.com	Pravibismane
Allvivo Vascular, Inc.	2000	USA	allvivo.com	Antimicrobial peptides
Sharklet Technologies, Inc.	2007	USA	sharklet.com	Engineered surfaces
Neem Biotech	1998	UK	neembiotech.com	Bio-active compounds from plant and marine sources
Bonalive Biomaterials Ltd	unknown	Finland	bonalive.com	Synthetic, osteoconductive material
Nobio	2015	Israel	nobio.com	Antimicrobial surface coatings
5D Health Protection Group Ltd	unknown	UK	5dhpg.com	Testing and consulting
Dermalink Technologies Inc.	unknown	USA	dermalinktech.com	Lauroyl arginine ethylester
Convatec	1978	UK	convatecgroup.com	Anti-biofilm wound dressings
NanoVibronix	2003	USA	nanovibronix.com	Low frequency, low intensity ultrasound acoustic waves
BIOVINC, LLC	unknown	USA	biovinc.com	Bisphosphonate bone targeting platform technology
Trellis Bioscience, Inc	1998	USA	trellisbio.com	Monoclonal antibodies; TRL1068, a high affinity mAb that targets the DNABII protein family
NovaFlux	unknown	USA	novaflux.com	Removal of biofilm from medical equipment
Innovotech	unknown	Canada	innovotech.ca	Biofilm screening assays and silver coatings
Ension, Inc.	2001	USA	ension.com	Non-fouling surfaces
N2 Biomedical, LLC	2013	USA	n2bio.com	Coatings and surface treatments for medical and dental applications
OneLife	unknown	Belgium	onelife-biofilmfree.com	Biofilm removal from medical devices
Imbed Biosciences	2010	USA	imbedbio.com	Silver-releasing wound dressing
Novabiotics	2004	UK	novabiotics.co.uk	Peptide and aminothiol based therapies
ContraFect	2008	USA	contrafect.com	Lysins, amurin peptides
Destiny Pharma	1996	UK	destinypharma.com	Novel antimicrobial nasal and dermal topicals
Eligo biosecience	2014	France	eligo.bio	Eligobiotics
Hyprotek	unknown	USA	hyprotekinc.com	Antimicrobial intravascular catheter (IVC) port cap
Amicoat	unknown	Norway	amicoat.com	Antimicrobial peptides

It is encouraging that so many companies are pursuing biofilm-related solutions for medical problems. As the importance of infectious disease continues to increase, with the growing problem of antimicrobial resistance (AMR), it is likely that innovative antimicrobials, including anti-biofilm therapeutics, will gain popularity and marketability. One issue complicating the matter is that the requirements for making a claim that a product is “anti-biofilm” is currently poorly defined. These requirements differ depending upon the FDA approval pathway, and also differ depending on country. Unlike the USDA where there are more clear-cut guidelines for standardized biofilm testing and precise terminology that can be used, FDA approvals of anti-biofilm claims have been issued on more of a case-by-case basis. Therefore, products may make claims of “controlling biofilm and microbial counts”, “penetrating and removing biofilm”, “helping prevent biofilm formation”, “biofilm removal and degradation”, “reduction of biofilm” or “inhibiting bacterial colonization”, without a standardized quantitative definition for what the claim means.

It is also encouraging that the term ‘biofilm’ is beginning to enter the popular vernacular, as evidenced by television commercials for toothpaste and mouthwash (among others) that refer to biofilm. This is a step forward because the more the general public understands biofilm and views it as a problem, the more likely there will be increased funding in this area. It is important though that we do not allow the unregulated, homeopathic-type, anti-biofilm products that are readily available online, to hijack the identity and muddle the importance of scientifically tested, FDA-approved anti-biofilm products. While there are clearly some natural compounds that have demonstrated clear anti-biofilm efficacy in peer-reviewed, published studies (most notably honey and various essential oils, e.g. Ref. [8,9]), a quick search for biofilm or anti-biofilm products online, results in a long list of very questionable products that make outrageous claims. It would be a pity if the commercial biofilm arena were overtaken by these types of products, which would likely sour the public image of the biofilm problem and result in a downward trend in funding over the long-term.

## Forecast for translation

So what does all of this information tell us about how well biofilm basic research is being translated from the bench-side to the bedside, and what does the forecast look like for future translation? According to the literature, anti-biofilm research still makes up a very small percentage of the total biofilm literature and it has only started to grow in the last 10 years. Therefore, it is not too surprising that the number of patents and clinical studies for anti-biofilm agents is relatively small. However, as the AMR problem increases, and people begin to focus more heavily on developing new drugs to fight infectious diseases, anti-biofilm agents are likely to gain more interest. Already in the U.S. we have seen FDA-approval requirements lowered for anti-infectives and government incentives given to companies and universities for the development of new drugs. It is also apparent from the current clinical trials that there is a focus on repurposing drugs that are already FDA-approved and testing drug combinations for anti-biofilm properties. So, this may be a branch of low hanging fruit that can easily be developed.

Surprisingly, although the scientific literature and experimental evidence surrounding anti-biofilm agents is quite small, there are a large number of companies selling products marketed to fight biofilm in some way. Industry interest in anti-biofilm products is promising and indicates that there is a significant commercial market for these products. However, it is important to keep in mind that requirements for making anti-biofilm claims are lax and inconsistent, so it is difficult to know how robustly these products have been tested. Also, as the biofilm concept continues to gain appreciation by the general public, we run the risk of having the area hijacked by pseudo-science and unproven products that make outrageous claims. This has already happened to a great extent in the microbiome area where pseudo-science books, products and companies offering services are prevalent. So it is important that biofilm researchers take a proactive role in providing outreach and imparting scientific knowledge to the public. Social media is key in this regard.

## How can we help at the bench-side?

Researchers and teachers have very important roles in shaping the future of the biofilm area. While biofilm research is rapidly growing, we need more translational studies! Researchers who are interested in translating their basic science discoveries into new anti-biofilm products should seek out collaborators who thoroughly understand the specific *in vivo* infection environment of interest. For example, if you are working on a new small molecule for preventing the establishment of *P. aeruginosa* biofilms in the lungs of cystic fibrosis patients, collaborate with pediatric pulmonologists or respiratory therapists, who see these patients and treat these infections on a regular basis. Learn more about the physiology and microenvironments present in the lung and use this information to refine your models so they are more clinically relevant.

For pre-clinical studies it is important to work on biofilms, but it should be in the ‘right’ environment, such as microcosm models tailored to a specific type of infection (e.g. sputum or wound media, or specialized cell culture models). *In vivo* animal models should also recapitulate the native infection as closely as possible, and use clinically relevant infecting doses and strains. When testing experimental antimicrobials and anti-biofilm agents there should be a high bar for efficacy, whether you are testing treatment of established infection or prophylaxis. A one-log reduction of bacterial load, likely means very little clinically when there are 10^8^ organisms left. Experimental agents should also be compared to standard of care treatments that are currently being used in the clinic. Clinical collaborators can provide important and current information about standard of care, clinical isolates and human samples to use in your studies, and patients for clinical trials. While proximity to clinical facilities can certainly be advantageous, many very successful basic science/clinical collaborations have been accomplished by investigators who are at different institutions. In fact, the biofilm field as a whole was pioneered by Bill Costerton, a basic scientist who sought out clinical collaborators from a wide area of specialties all over the world. His efforts were followed by many other great biofilm ambassadors, such as Mark Shirtliff, Paul Stoodley and Thomas Bjarnsholt who have successfully pursued multi-disciplinary projects that bring together engineers, microbiologists, physicists, mathematicians and clinicians to solve biofilm problems.

Industrial collaborations can also be mutually advantageous. It is often small start-up companies and University-initiated incubator companies who are attempting to develop anti-biofilm products on shoestring budgets. Many of these companies lack sufficient research and development facilities in which to test their agents. We can support their efforts by serving as scientific advisors, helping design robust pre-clinical studies, and collaborating to test their products in our laboratories, using proper models and experimental design. These collaborations not only help companies advance their products, but can provide biofilm researchers with insight into the commercialization process, and a network of industry connections that could help them in future developmental endeavors.

Lastly, efforts in teaching biofilm theory and methods are needed. While courses on biofilm are becoming more common at undergraduate institutions, this content is frequently missing from the curriculums of professional schools. For example, the curriculums of many medical, nursing, pharmacy and allied health schools are completely lacking any biofilm content at all. Unfortunately, this results in health professionals entering clinical duty with no knowledge about biofilms or the challenges they represent. The same is true for some clinical laboratory science programs, resulting in clinical microbiologists performing susceptibility testing of clinical isolates for hospitals, who do not understand how biofilms can impart antibiotic tolerance.

For many health professions programs and medical schools, the curricular content is structured around the information needed for students to pass accreditation exams. Therefore, one solution to solve these deficiencies would be to pressure accreditation bodies to add biofilm content to their exams. In addition to the need for more biofilm-related medical education, we also need effective biofilm ambassadors who can convey the importance of biofilm to different specialties. This might include attending and presenting at conferences that are specifically geared to clinical lab sciences, nursing care, respiratory therapists, or wound care, for example.

Overall, the future for anti-biofilm therapeutics is quite promising. The data discussed above indicate an upward trend in translational studies, a clear commercial interest, and a definite medical need. Given these current indicators, both the biofilm field in general and the funding for biofilm research will continue to grow. The biofilm field also has the benefit of an extremely enthusiastic and collaborative, global group of scientists who are highly motivated to expand and evolve the research. This is both a testament to the pioneers of the field and the ongoing hard work of current leaders and mentors.

## CRediT authorship contribution statement

**Kendra P. Rumbaugh:** Conceptualization, Data curation, Formal analysis, Writing - original draft, Writing - review & editing.

## Declaration of competing interest

The authors declare that they have no known competing financial interests or personal relationships that could have appeared to influence the work reported in this paper.
